# Linking Stoichiometric Homeostasis of Microorganisms with Soil Phosphorus Dynamics in Wetlands Subjected to Microcosm Warming

**DOI:** 10.1371/journal.pone.0085575

**Published:** 2014-01-27

**Authors:** Hang Wang, HongYi Li, ZhiJian Zhang, Jeffrey D. Muehlbauer, Qiang He, XinHua Xu, ChunLei Yue, DaQian Jiang

**Affiliations:** 1 College of Natural Resource and Environmental Sciences, China Academy of West Development, Zhejiang University, Hangzhou, Zhejiang Province, China; 2 Curriculum for the Environment & Ecology, University of North Carolina at Chapel Hill, Chapel Hill, North Carolina, United States of America; 3 Department of Civil & Environmental Engineering, University of Tennessee, Knoxville, Tennessee, United States of America; 4 Institute of Ecology, Zhejiang Forestry Academy, Hangzhou, China; 5 Department of Earth and Environmental Engineering, Henry Krumb School of Mines, Columbia University, New York, New York, United States of America; Centrum Wiskunde & Informatica (CWI) & Netherlands Institute for Systems Biology, Netherlands

## Abstract

Soil biogeochemical processes and the ecological stability of wetland ecosystems under global warming scenarios have gained increasing attention worldwide. Changes in the capacity of microorganisms to maintain stoichiometric homeostasis, or relatively stable internal concentrations of elements, may serve as an indicator of alterations to soil biogeochemical processes and their associated ecological feedbacks. In this study, an outdoor computerized microcosm was set up to simulate a warmed (+5°C) climate scenario, using novel, minute-scale temperature manipulation technology. The principle of stoichiometric homeostasis was adopted to illustrate phosphorus (P) biogeochemical cycling coupled with carbon (C) dynamics within the soil-microorganism complex. We hypothesized that enhancing the flux of P from soil to water under warming scenarios is tightly coupled with a decrease in homeostatic regulation ability in wetland ecosystems. Results indicate that experimental warming impaired the ability of stoichiometric homeostasis (*H*) to regulate biogeochemical processes, enhancing the ecological role of wetland soil as an ecological source for both P and C. The potential P flux from soil to water ranged from 0.11 to 34.51 mg m^−2^ d^−1^ in the control and 0.07 to 61.26 mg m^−2^ d^−1^ in the warmed treatment. The synergistic function of C-P acquisition is an important mechanism underlying C∶P stoichiometric balance for soil microorganisms under warming. For both treatment groups, strongly significant (*p*<0.001) relationships fitting a negative allometric power model with a fractional exponent were found between n-*H_C∶P_* (the specialized homeostatic regulation ability as a ratio of soil highly labile organic carbon to dissolved reactive phosphorus in porewater) and potential P flux. Although many factors may affect soil P dynamics, the n-*H_C∶P_* term fundamentally reflects the stoichiometric balance or interactions between the energy landscape (i.e., C) and flow of resources (e.g., N and P), and can be a useful ecological tool for assessing potential P flux in ecosystems.

## Introduction

The principle of stoichiometric homeostasis states that organisms maintain relatively stable levels of biologically-relevant elements (e.g., carbon, nitrogen, and phosphorous) over time. This concept is based on ecological stoichiometry and is primarily applied to trophic interactions [1]. By modulating organism responses to key environmental drivers (e.g., nutrient fertilization), stoichiometric homeostasis is also a major mechanism responsible for the structure, function, and stability of ecosystems. Therefore, deviations from stoichiometric homeostasis can serve as indicators of environmental fluctuations that may be useful in ecological evaluation. The homeostatic regulation coefficient (*H*), which may be accessed through a regression model (equation 1, in the Materials and Methods) is a measure of homoeostatic regulation ability and is commonly used as an indicator of these ecological changes [2].

The average global surface temperature has increased by 0.74°C since 1850 and is likely to increase an additional 1.1–6.4°C by the end of this century [3], [4]. Temperature is one of the primary determinants affecting the metabolic rates of organisms, from cells to global-scale ecosystems, based on the metabolic theory of ecology [5], [6]. Even a slight increase in temperature can alter energy flow and resource cycles in ecosystems and may affect environmental quality [3], [7]. For instance, global soil respiration increased by 0.1 Pg yr^−1^ between 1989 and 2008 due to a high temperature anomaly [8]. Understanding the response of stoichiometric homeostasis to global warming and its associated ecological feedback to the biosphere is gaining an increasing degree of attention worldwide.

Studies of stoichiometric homeostasis have strongly emphasized physiological variation in elemental composition in macroscopic aquatic species [9]. To a lesser extent, some studies have also quantified the degree of stoichiometric homeostasis in terrestrial plants [2], [10], [11]. Stoichiometric homeostasis investigations in soil microorganisms subjected to environmental changes, however, are rare. Biogeochemical processes at the soil-water interface play a fundamental role in overall soil development, and they are the primary driving force behind key ecosystem functions such as community productivity and water quality [12], [13]. Although stoichiometric homeostasis reflects the net outcome of many underlying physiological and biochemical adjustments as organisms respond to their surroundings [14], to our knowledge, few studies have linked any change in stoichiometric homeostasis to biogeochemical processes in a soil ecosystem affected by global warming.

Wetlands are one of the most productive and biologically diverse ecosystem types on the planet, where the chemical and, in particular, nutrient composition of shallow groundwater and surface water can be altered by a range of biogeochemical processes [15]. Many of the elements and nutrients that cycle through wetlands have ecosystem and global-scale effects; for example, phosphorous (P), is the key element affecting eutrophication in aquatic ecosystems [16], limiting coastal ecosystem processes [17], and in regulating allometry in soil food webs [18]. However, global warming may impact the inherent biogeochemical balance of P and other major elements. Diffusive models predict that elemental fluxes of P and nitrogen (N) to receiving waters are more than 2 times higher during summer (i.e., when temperatures are warmer) than in other seasons [19], [20]. Further, an 18-month microcosm investigation using a minute and seasonal scale temperature manipulation verified that warming induced substantial mobilization of P from wetland sediment to water [21], [22]. In other studies, excessive P loading under high temperature accelerated the risk of eutrophication in receiving waters [15], [20], [23] and altered carbon (C) assimilation and N accumulation in aquatic plants [24]. These studies suggest that warming or increasing temperature disturbs P equilibrium between sediment and water. However, the link between stoichiometric homeostasis and P dynamics in wetland soil subjected to global warming is poorly understood, which impedes the development of ecological regulatory strategies for coping with global warming scenarios. Additionally, wetlands play a vital role in the global C cycle and may respond strongly to climate change [25]. This is important because the flow of C energy and material also controls the biogeochemical cycling of many other elements in Earth's ecosystems [5], [26], [27], [28]. Thus, integrating C∶P stoichiometry is vital to understanding the connection between soil P dynamics and the regulation of stoichiometric homeostasis in wetland ecosystems.

The objective of our study was to shed new light on P dynamics in response to global warming, from the perspective of C∶P stoichiometric homeostasis in six subtropical wetlands located in the Yangtze River delta in southeast China. We simulated global warming using a novel, in situ deployment of a microcosm device that mimics a warming scenario of 5°C above ambient temperature. The link between potential P flux from soil to water and the homeostatic regulation ability of soil microorganisms was also examined. We hypothesized that enhancing P flux from soil to water under warming scenarios is tightly coupled with a decrease in homeostatic regulation ability in wetland ecosystems.

## Materials and Methods

This work is unrelated to any ethics issues. No specific permit was required for the described field study because the sampling locations were not located in protected areas or private land. The experimental field studies did not involve endangered or protected species.

### Study sites

The study sites (120°41′31″E, 30°53′55″N to 120°33′32″E, 30°01′58″N) were located in the southern region of the Taihu Lake Basin and the NingShao Plain within the Yangtze River delta in southeast China. The climate in this area is subtropical monsoon with an annual average rainfall of 1350 mm and an annual average temperature of 26°C in summer and 4°C in winter.

The Supporting Information contains details about the six sites related to geographical position, hydrological parameters, and dominant vegetation during sampling (Table S1), as well as the physico-chemical properties of the six collected soils (Table S2). In brief, YaTang riverine wetland (YT) is in an advanced state of eutrophication and is classified as eutrophic, with the highest P and C total soil content among the six study sites. Soils sampled from XiaZhuhu wetland (XZ), XiXi National Wetland Park (XX), BaoYang riverine wetland (BY), and JinHu wetland (JH) are in a meso-eutrophic state, while the lowest soil C and P content among the sites was found in ShiQiu multipond wetland (SQ). Because these selected study sites represented the range of typical soil C and P conditions across subtropical wetland ecosystems in southeast China, we did not design any additional C or P treatments, which would have required artificial manipulations of the soil concentrations of these elements.

### Microcosm configuration and sampling regime

A custom-built, novel microcosm (Fig. S1) simulating climate warming was developed under both present-day ambient temperature conditions (Control) and simulated warming conditions of 5°C above ambient temperature (Warmed). Details on the configuration of this microcosm system and its operation were either described previously [21] or can be found in Text S1. Compared to fixed-temperature laboratory incubations, this novel microcosm offers higher temporal resolution temperature control (on a minute scale), which simulates more realistic warming conditions.

Details on the establishment of wetland columns and water-soil samplings during the microcosm investigation are provided in Text S2. Briefly, each wetland column consisted of a prefabricated PVC pipe assembly (45.0 cm in height and 10.0 cm in internal diameter) that was designed to hold 20 cm of fresh soil and 20 cm of corresponding overlying water. Columns were installed in May 2008, and three replicates were placed inside each of the two incubation boxes. In this study, three samplings of water (overlying water and porewater) and 0–5 cm of topsoil were carried out in July and November 2010 and March 2011, which were used to illustrate the link between soil microorganism stoichiometric homeostasis and soil P dynamics in wetlands subjected to experimental warming.

### Water and soil analysis

All water and soil samples were frozen at −15°C prior to analysis. After thawing, water was filtered through a 0.45-µm filter. Phosphorous in filtered samples was measured using a continuous flow analyzer (Autoanalyzer III, Bran+Luebbe, Germany) with a spectrophotometer set at 880 nm (Murphy and Riley, 1962). This is the absorbance of dissolved reactive phosphorus (DRP), which represents the bioavailable P fraction for microorganisms and/or algae in surface water. Porewater samples for dissolved organic carbon (DOC) analysis were first acidified (10% HCl) and purged with inert gas to remove any inorganic carbon, then analyzed using a Shimadzu TOC 5000 analyzer (Shimadzu Scientific Instruments, Columbia, Maryland, USA).

After thawing, soil samples were analyzed for C and P in microbial biomass (Table 1) using the fumigation-extraction method described by Wu et al. [29] and Brookes et al. [30]. In brief, soil C and P in fumigated and non-fumigated soil samples was extracted using solutions of 0.5 mol L^−1^ K_2_SO_4_ and 0.5 mol L^−1^ NaHCO_3_. The differences in extractable C and P between fumigated and non-fumigated soil were assumed to be released from lysed soil microbes, i.e., microbial biomass C and P (MB-C, MB-P). By utilizing the susceptibility of organic C to KMnO_4_ oxidation [31], three fractions of labile organic C in these wetland columns; namely, highly labile organic C (HLOC), mid-labile organic C (MLOC) and labile organic C (LOC), were determined using 33 mmol L^−1^, 167 mmol L^−1^, and 333 mmol L^−1^ of KMnO_4_, respectively.

**Table 1:** Concentrations of soil microbial biomass (MB; MB-C: microbial carbon; MB-P: microbial phosphorus), soil carbon (TOC: total organic carbon; HLOC: highly labile organic carbon; MLOC: mid-labile organic carbon; and LOC: labile organic carbon), and carbon in porewater (DOC: dissolved organic carbon) in wetland columns in the microcosm experiment (Control: ambient temperature; Warmed: ambient temperature +5°C).

Treatment		Soil microbial biomass				Soil carbon				DOC in porewater(mg kg^−1^)
		Total mass (mg g^−1^)	MB-C (mg g^−1^)	MB-P (mg kg^−1^)	Microbial C∶P stoichiometric ratio	TOC (g kg^−1^)	HLOC (mg g^−1^)	MLOC (mg g^−1^)	LOC (mg g^−1^)	
JH	Control	0.402±0.023	0.386±0.022	15.0±2.3	68.6∶1	25.6±1.4	2.28±0.09	2.75±0.66	3.75±0.71	13.2±2.3
	Warmed	0.493±0.010	0.477±0.008	16.1±2.1	79.0∶1	22.3±2.1	2.31±0.37	3.16±0.70	5.48±1.31	16.6±3.1
*p*		0.064	**0.050**	0.568		**0.028**	0.899	0.498	0.113	0.182
XZ	Control	1.12±0.02	1.08±0.02	25.9±2.4	111∶1	54.8±5.9	5.78±0.31	8.55±0.29	9.93±0.43	17.3±0.9
	Warmed	1.47±0.03	1.42±0.04	33.4±2.3	113∶1	49.2±2.6	6.35±0.06	8.67±0.13	9.93±0.32	21.6±2.2
*p*		**<0.001**	**0.009**	**0.005**		0.054	0.055	0.575	0.990	**0.038**
YT	Control	1.42±0.01	1.37±0.01	36.4±2.9	100∶1	133±10	9.13±0.23	13.0±1.0	18.9±4.2	20.2±3.3
	Warmed	1.73±0.06	1.66±0.04	46.1±5.7	96.0∶1	117±3	10.9±0.1	18.6±0.9	20.5±4.3	22.7±9.9
*p*		**0.001**	**0.035**	**0.045**		**0.024**	**<0.001**	**0.002**	0.681	0.701
XX	Control	1.01±0.03	0.964±0.024	33.4±4.4	77.0∶1	35.6±1.7	2.14±0.07	3.12±0.23	3.70±0.43	16.2±3.0
	Warmed	1.31±0.08	1.25±0.11	42.0±2.3	79.4∶1	31.8±1.3	3.39±0.06	3.65±0.19	4.32±0.83	24.3±8.7
*p*		**0.003**	0.067	**0.028**		**0.011**	**<0.001**	**0.026**	**0.039**	0.208
BY	Control	1.07±0.06	1.04±0.06	34.5±2.4	80.4∶1	37.6±1.9	2.90±0.12	3.98±0.05	4.50±0.09	18.6±1.5
	Warmed	1.13±0.07	1.10±0.06	36.6±2.6	80.1∶1	32.4±2.1	3.59±0.36	5.91±0.16	7.06±0.33	21.8±1.0
*p*		0.299	0.317	0.435		**0.043**	**0.033**	**<0.001**	**<0.001**	**0.027**
SQ	Control	0.183±0.018	0.179±0.017	4.25±0.87	112∶1	14.2±2.3	0.556±0.025	0.889±0.673	1.73±0.33	8.14±0.96
	Warmed	0.469±0.011	0.456±0.013	13.5±2.7	90.1∶1	15.3±1.7	2.61±0.05	3.74±0.15	4.23±0.16	12.3±1.2
*p*		**<0.001**	**<0.000**	**0.005**		0.443	**<0.001**	**0.002**	**<0.001**	**0.006**

*p*-values refer to significant differences between two treatments. Sampling date is November 2010.

### Calculation of homeostatic regulation ability of soil microorganisms

The homeostatic regulation ability of soil microorganisms was estimated by calculating the homeostatic regulation coefficient (*H*) according to the following equation [1]:
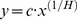
(1)where *y* is the microbial C or P concentration (mg g^−1^ or mg kg^−1^ of dry soil weight) or the C∶P molar ratio of soil microorganisms; *x* is the organic C concentration (including HLOC, MLOC, and LOC) in soil, DRP or DOC concentration in porewater, or the C∶P molar ratio among the soil organic C and porewater DRP; and *c* is a constant. *H* is defined as the homeostatic regulation ability of soil microorganisms; as the value of *H* increases, the ecosystem trends toward a higher degree of ecological stability and resilience. For example, higher values of *H_N_* and *H_P_* in grasses receiving field N and P fertilizations were significantly associated with higher species dominance and stability [2]. According to ecological allometry [5], [32], *H* may be classified as an equilibrium constant with respect to functions of organism metabolic and stoichiometric processes.

The first derivative of regression equation (1), reflecting the response rate of *y* to *x*, is given as follows:
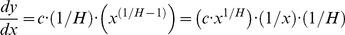
(2)By substituting in variables from equation (1), equation (2) can be transformed as:
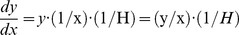
(3)Here, the value of (*y/x*) is defined as the specific variable for 1/*H,* which may be transformed into (*x/y*) responding to *H*. Therefore, we define n-*H_i_* as the specialized homeostatic regulation ability for each of the tested wetland soil columns:
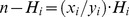
(4)where *x_i_* is the measured value of the organic C concentration (including HLOC, MLOC, and LOC) in soil, DRP or DOC concentration in porewater, or the C∶P molar ratio among the soil organic C and porewater DRP for each soil sample; *y_i_* is the microbial C or P concentration (mg g^−1^ or mg kg^−1^ of dry soil weight) or the C∶P molar ratio of soil microorganisms for each soil sample; *i* is the type of parameter (form of HLOC, MLOC, LOC, DRP, DOC, or ratio of C∶P for soil or soil microbes); and *H* refers to the homeostatic regulation coefficient calculated by equation (1) according to the respective for each of the two treatments (Control and Warmed) in terms of the *i* parameter.

Potential P flux (F_P_, mg m^−2^ d^−1^) was used to evaluate the potential P transfer out of porewater under experimental warming according to the following equation:
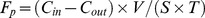
(5)where *C_in_* *C_in_* is the DRP concentration in the porewater (mg L^−1^), *C_out_* is the P concentration in the overlying water (mg L^−1^), *V* is the volume of overlying water (mL), *S* is the area of the wetland column (m^2^) and *T* is the interval between two sampling dates (d). Due to the relatively static hydrological conditions in the wetlands (Table S1), DRP was the most important form for P exchange between soil and water in the study. Soil microorganisms are also more sensitive to DRP than to TP due to its bioavailability. Therefore, values of F_P_ (and n-*H_i_*) were evaluated by the form of DRP rather than TP. The potential P flux defined here provides a good indicator for assessing P concentration gradients between overlying water and porewater, with high values suggesting a high risk of P transfer from soil to overlying water.

In this study, aquatic macrophytes (e.g., *Phragmites communis*) were temporarily excluded from soil columns, but small benthic organisms (e.g., *Margarya melanoide*) and plankton (e.g., *Spirogyra*) were preserved. Data collected from water and soil samples were expressed as the mean plus standard error. Paired Student's *t*-tests were used to compare the effects of warming on soil microorganism and C fractions, P concentrations in porewater and overlying water, and potential P flux. Two-way analysis of variance (repeated ANOVA) tests were carried out using SPSS software (version 15.0) to examine the effects of experimental warming, sampling time, and their interaction on soil biochemical traits and potential P flux. Statistical test results were considered significant at the *p*<0.05 level.

## Results

### Soil carbon and phosphorus potential flux in response to experimental warming

After >2.5 y of incubation (in Nov 2010), experimental warming increased soil microbial biomass, soil labile C, and porewater DOC (Table 1); however, warming decreased concentrations of total soil C at all wetlands except site SQ. Meanwhile, although fluctuations in DRP concentrations were found in the overlying water, DRP in porewater increased in the warmed treatment by 52% to 137% compared to the ambient treatment, except in the SQ wetland (Table 2). On average, potential P flux in the six wetlands ranged from 0.11 to 34.51 mg m^−2^ d^−1^ in the control and 0.07 to 61.26 mg m^−2^ d^−1^ in the warmed treatment (Table 2). Two-way analysis of variance indicated that experimental warming significantly increased potential P flux, except in the SQ wetland. There were also significant differences in soil C pools and potential P flux between Warmed and Control treatments (Table 3). A significant interaction between treatment and season (sampling date) was found for soil C pools and potential P flux as well, although there were no significant differences in these variables by season alone.

**Table 2:** Phosphorus (P) concentrations in overlying water and porewater, and the potential flux of dissolved reactive P in wetland columns in the microcosm experiment (Control: ambient temperature; Warmed: ambient temperature +5°C).

Wetland column	Treatments	Overlying water (mg L^−1^)	Porewater (mg L^−1^)	Potential P flux (mg m^−2^ d^−1^)
	July 2010			
JH	Control	0.066±0.038	0.063±0.005	0.21±0.07
	Warmed	0.017±0.009	0.095±0.001	0.32±0.09
*p*		**0.022**	0.004	**0.018**
XZ	Control	0.070±0.026	0.266±0.003	0.90±0.03
	Warmed	0.024±0.024	0.612±0.063	2.50±0.38
*p*		**0.012**	**0.039**	**0.003**
YT	Control	1.507±1.22	2.334±0.703	15.30±3.56
	Warmed	0.909±0.51	4.537±1.510	35.26±4.61
*p*		0.304	**0.002**	**<0.000**
XX	Control	0.106±0.076	0.330±0.015	2.14±0.17
	Warmed	0.038±0.031	0.570±0.033	3.65±0.10
*p*		0.091	**0.023**	**0.004**
BY	Control	0.063±0.061	0.104±0.001	0.61±0.02
	Warmed	0.051±0.079	0.231±0.001	0.98±0.24
*p*		0.783	**<0.000**	**0.017**
SQ	Control	0.016±0.008	0.088±0.015	0.11±0.02
	Warmed	0.012±0.010	0.021±0.009	0.08±0.01
*p*		0.431	0.025	0.412
	March 2011			
JH	Control	0.018±0.000	0.182±0.102	0.63±0.37
	Warmed	0.016±0.000	0.354±0.048	1.07±0.23
*p*		0.467	**0.016**	**0.004**
XZ	Control	0.029±0.014	0.720±0.286	2.10±0.13
	Warmed	0.018±0.000	1.704±0.189	4.36±0.78
*p*		0.187	**0.013**	**0.006**
YT	Control	0.042±0.008	5.83±1.23	34.51±10.36
	Warmed	0.047±0.012	8.34±0.41	61.26±6.12
*p*		0.572	**0.011**	**<0.000**
XX	Control	0.016±0.006	1.17±0.18	3.38±0.71
	Warmed	0.013±0.004	2.53±0.34	11.72±2.10
*p*		0.320	**0.004**	**<0.000**
BY	Control	0.016±0.000	0.390±0.067	1.06±0.12
	Warmed	0.022±0.000	0.653±0.182	2.38±0.53
*p*		**0.012**	**0.039**	**0.034**
SQ	Control	0.012±0.001	0.068±0.019	0.16±0.09
	Warmed	0.016±0.000	0.044±0.040	0.07±0.03
*p*		0.490	0.193	0.387

*p*-values refer to significant differences between two treatments.

**Table 3:** Results of two-way analysis of variance (repeated ANOVA) showing the *p*-values for soil microorganisms and soil carbon and potential phosphorus (P) flux under experimental warming and sampling time. MB-C: microbial carbon, MB-P: microbial phosphorus, TOC: total organic carbon, HLOC: highly labile organic carbon, MLOC: mid-labile organic carbon, and LOC: labile organic carbon.

Factor	Soil microorganisms			Soil carbon				Potential P flux (mg m^−2^ d^−1^)
	Total (mg g^−1^)	MB-C (mg g^−1^)	MB-P (mg kg^−1^)	TOC (g kg^−1^)	HLOC (mg kg^−1^)	MLOC (mg kg^−1^)	LOC (mg kg^−1^)	
Treatment	**0.007**	**0.005**	**0.008**	**0.013**	**0.011**	**0.038**	**0.007**	**0.033**
Time	0.285	0.321	0.308	0.079	0.563	0.439	0.407	0.185
Treatment×Time	**0.017**	**0.016**	**0.009**	**0.006**	**0.019**	0.076	**0.016**	**0.047**

*p*-values smaller than 0.05 are in bold to indicate statistical significance.

### Microorganism stoichiometric homeostasis in response to experimental warming

**Figure 1:**
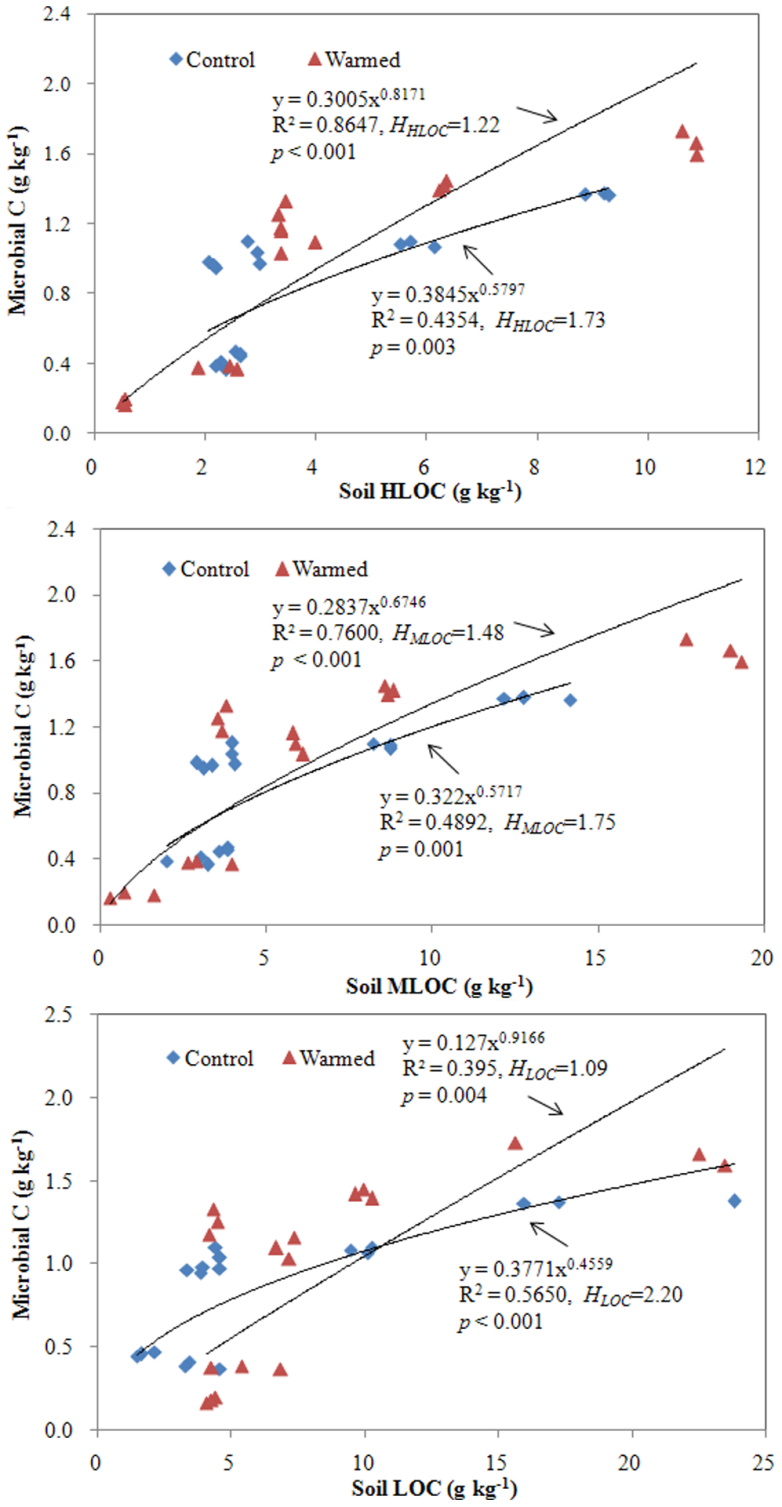
Relationships between soil microbial carbon (MB-C) and soil organic carbon (in forms of HLOC: highly labile organic carbon, MLOC: mid-labile organic carbon, and LOC: labile organic carbon). Control and Warmed represent treatments of ambient temperature and ambient temperature +5°C, respectively. Sampling date is March 2011. Coefficients fitting the data to the equation for stoichiometric homeostasis are given along with an estimate of *H_C_*.

**Figure 2:**
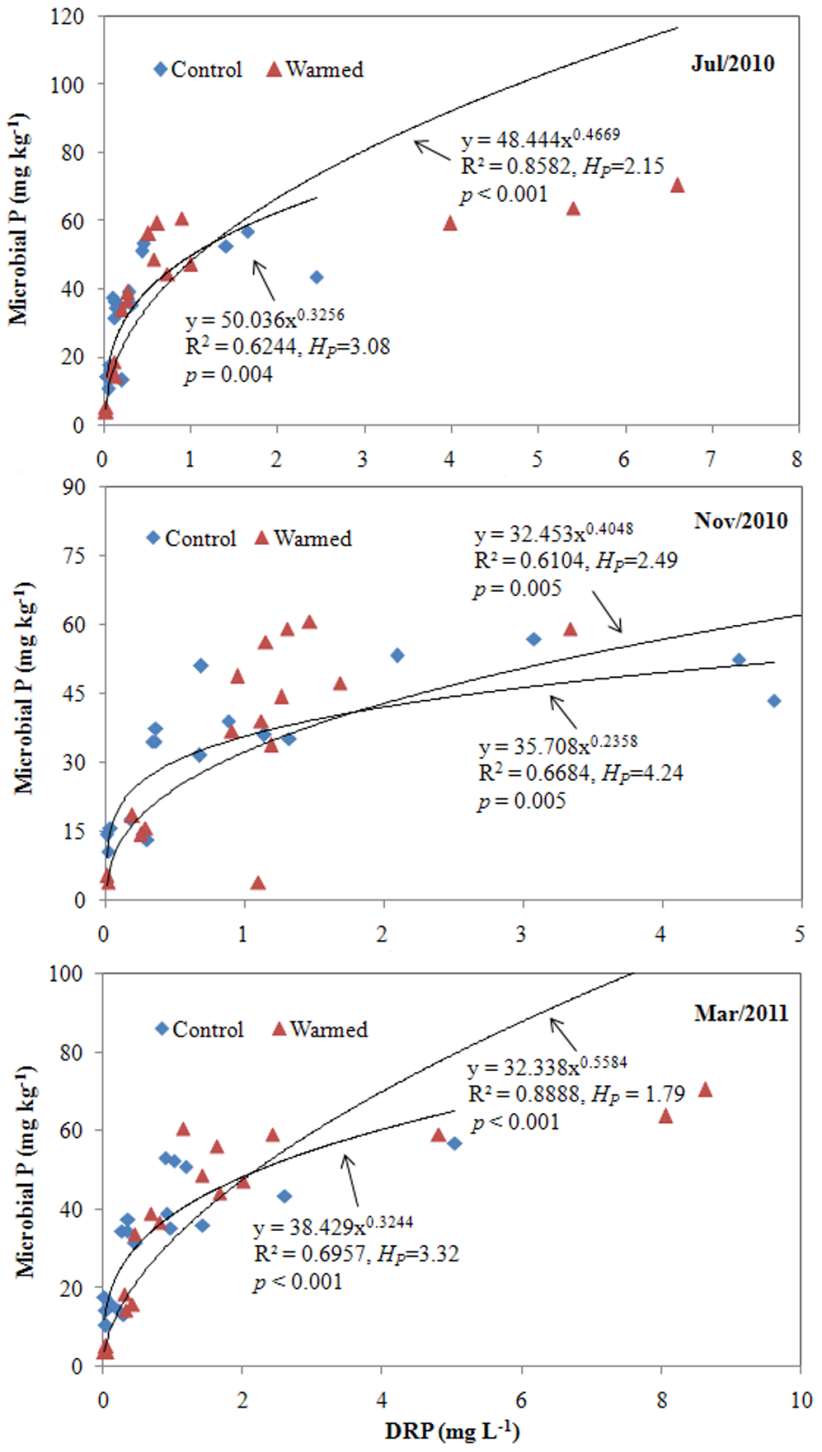
Relationships between soil microbial phosphorus (MB-P) content and dissolved reactive phosphorus (DRP) in porewater. Control and Warmed represent treatments of ambient temperature and ambient temperature +5°C, respectively. Coefficients fitting the data to the equation for stoichiometric homeostasis are given along with an estimate of *H_P_*.

**Figure 3:**
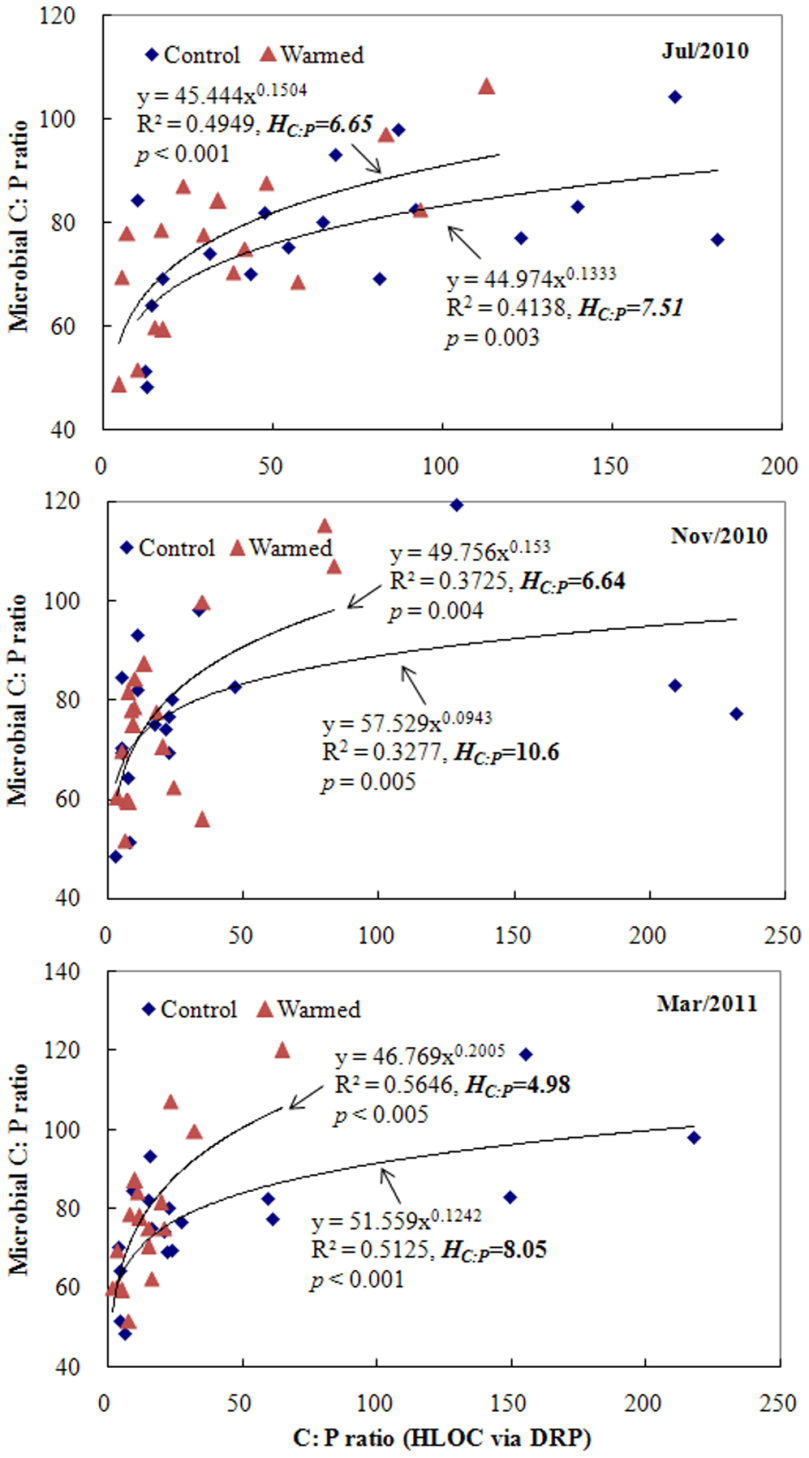
Relationships between the molar ratios of carbon to phosphorus (C∶P) in soil microbial biomass and the C∶P ratios of soil highly labile organic carbon (HLOC) to dissolved reactive phosphorus (DRP) in porewater. Control and Warmed represent treatments of ambient temperature and ambient temperature +5°C, respectively. Coefficients fitting the data to the equation for stoichiometric homeostasis are given along with an estimate of *H_C∶P_*.

The relationships between soil microorganism biomass and three forms of soil carbon concentrations (HLOC, MLOC, and LOC) sampled in Mar. 2011 were rigorously and significantly described by the stoichiometric homeostasis model (Fig. 1, *p*<0.01). The values of *H* in the warmed treatment were lower than those in the control, with *H* values of 1.22 and 1.73 for HLOC, 1.48 and 1.75 for MLOC, and 1.09 and 2.20 for LOC in warmed and control treatments, respectively (Fig. 1). Results from the other two samplings (data not shown) were fundamentally similar to the Mar. 2011 sampling. There was no consistent pattern in the homeostasis model fit between DOC and microorganism C among the three samplings (Table 4). However, strongly significant and positive correlations between DRP in porewater and soil microorganism biomass P fit a power model for these three consecutive samplings (Fig. 2), with lower values of *H* under the warmed treatment (1.79 to 2.49) compared to the control (3.08 to 4.24). Although no significant difference between these two treatments was found for the C∶P stoichiometric ratios of soil microorganisms (paired *t*-test *p* = 0.607, Table 1), strongly significant correlations existed between soil C∶P ratios of HLOC to DRP and soil microorganism C∶P for these three samplings under the two treatments (Fig. 3). More importantly, the C∶P ratio-related *H* values (i.e., *H_C∶P_*) obtained from the warmed group were consistently lower than those from the control group.

**Table 4:** Homeostatic regulation coefficient (*H*) in soils collected from wetland columns in the microcosm experiment (Control: ambient temperature; Warmed: ambient temperature +5°C).

Variable	Dependent variable	Sampling data	Control group			Warmed group		
			*H*	R^2^	*p*	*H*	R^2^	*p*
Porewater DOC	Microbial biomass C	Jul 2010	−5.00	0.084	0.203	4.26	0.085	0.219
		Nov 2010	2.76	0.274	**0.004**	3.00	0.257	**0.008**
		Mar 2011	1.71	0.386	**0.000**	−20.0	0.019	0.452
Porewater DOC∶DRP	Microbial C∶P	Jul 2010	500	0.014	0.672	100	0.005	0.953
		Nov 2010	250	0.000	–	71.4	0.002	0.976
		Mar 2011	55.6	0.007	0.872	43.4	0.016	0.553
Soil LOC: porewater DRP	Microbial C∶P	Jul 2010	6.76	0.303	**0.004**	9.43	0.463	**0.000**
		Nov 2010	12.9	0.275	**0.003**	13.7	0.237	**0.010**
		Mar 2011	12.6	0.154	**0.013**	7.46	0.545	**0.000**
Soil MLOC: porewater DRP	Microbial C∶P	Jul 2010	8.26	0.340	**0.003**	6.90	0.391	**0.000**
		Nov 2010	16.1	0.268	**0.008**	22.2	0.071	0.237
		Mar 2011	12.8	0.213	**0.013**	5.18	0.461	**0.000**

*p*-values refer to significant differences between two treatments. DOC and DRP refer to dissolved organic carbon and dissolved reactive phosphorus in porewater while MLOC and LOC refer to mid-labile organic carbon and labile organic carbon in soil, respectively.

**Figure 4:**
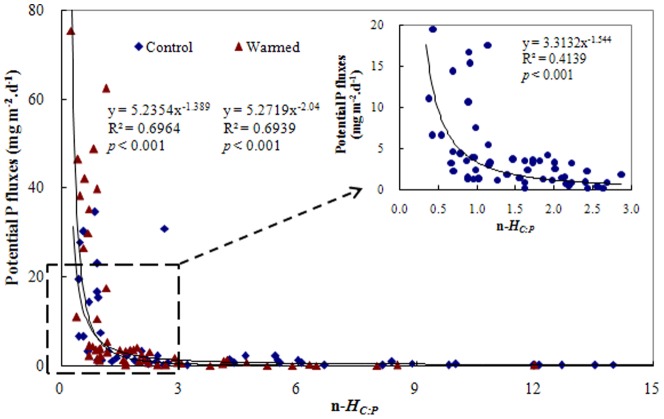
Relationship between potential phosphorus (P) flux and the specialized ability of stoichiometric homeostasis (n-*H_C∶P_*) as a molar ratio of soil highly labial organic carbon (HLOC) to dissolved reactive phosphorus (DRP) in each of the wetland soil columns. Control and Warmed represent treatments of ambient temperature and ambient temperature +5°C, respectively. The data for P flux and n-*H_C∶P_* less than 20 mg m^−2^ d^−1^ and 3.0, respectively, are plotted in the figure inset.

No significant relationship was found between porewater ratios of DOC∶DRP and soil microbial C∶P (Table 4). Relationships were observed for other comparisons of soil LOC to porewater DRP and soil MLOC to porewater DRP vs soil microorganism C∶P ratios (Table 4); however, among the three samplings, corresponding *H_C∶P_* values showed no consistent trends between the two treatment groups. This is in contrast to soil HLOC to porewater DRP results, in which a consistent increase in *H_C∶P_* was observed from control to warmed treatments across all three samplings (Fig. 3). Specific n-*H_C∶P_* values for each wetland column and soil treatment (Warmed or Control) were subsequently calculated using the *H_C∶P_* values derived from these HLOC∶DRP regressions, using C∶P ratios (HLOC∶DRP) in porewater as the ‘*x_i_*’ variable and soil microbial C∶P ratios as the ‘*y_i_*’ variable in equation (4). Again, strongly significant (*p*<0.001) linkages that fit a power model between n-*H_C∶P_* and potential P flux (i.e., allometric relationships [5]) were found for both treatment groups (Fig. 4). Notably, these two fitted curves strongly overlapped, and the regression equation for the two datasets combined (*y* = 5.261*×* ^−1.7509^, R^2^ = 0.5893) was almost identical to the equations derived from each dataset individually. The data for potential P flux and n-*H_C∶P_* values were concentrated in ranges less than 20 mg m^−2^ d^−1^ and 3.0, respectively (Fig. 4).

## Discussion

### Does homeostasis relate to the ecological role of soil in C and P cycling?

The tight linkages in this study between different pools of soil labile C and soil microbial C (Fig. 1), and porewater DRP and soil microbial P (Fig. 2) fit the stoichiometric homeostasis model. This supports the view that stoichiometric homeostasis is an important mechanism underpinning the dynamics of C and P resource availability at the soil-microbe interphase in wetland ecosystems. It confirms that the composition of C and P in microbial assemblages is closely related to resource availability in ecosystems [33], [34], as soil microorganism C and P increases were related to the increases in soil C and P availability observed in the warmed treatment in this study (Table 1 & 2). Based on the principle of homeostatic regulation ability [1] and its prior application to a grassland ecosystem [2], decreased *H* values for soil labile C and DRP in warmed columns indicate that experimental warming impaired the ability of soil organisms to regulate C and P biogeochemical turnover in the studied wetland ecosystems. This loss of homoeostatic regulation ability is consistent with the observed decrease in soil TOC (Table 1) and the increase in potential P flux (Table 2). Comparison of the data for the decrease in soil TOC and the increase in both soil labile C and microorganism C (Table 1) further indicates that losses of total organic C in warmed soils were mainly derived from non-active C pools; i.e., the recalcitrant organic C. Recalcitrant organic fractions generally represent a large proportion of the total C pool and have relatively slow turnover rates [35], [36], and generation of recalcitrant organic matter is regarded as a vital process for C sequestration [36]. Excluding P that is assimilated into microbial biomass (Table 1), warming subjects the remaining P pool to increases in enzymatic biodegradation of organic phosphorus [21], P desorption [20], [37], and inorganic P solubilization [38], which could enhance the strength of potential P flux (Table 2) as well as increase DRP in porewater. The ecological role of wetland soil as a source for C and P, coupled with lower *H* values observed in the warming treatment compared to the control, suggests that *H* may yield valuable insight into the ecological role of soil in C and P biogeochemical turnover and its response to global warming.

### Does homeostasis relate to stoichiometric balance?

Although experimental warming did affect the soil microbial C∶P stoichiometric ratios for particular samples to some degree (Table 1), no overall significant difference was found between the two (Warmed and Control) groups. However, C∶P stoichiometric ratios in the soil microbial community did seem to vary according to geographical (biotic and abiotic) features [39] across the six wetlands (Table 1 and Fig. 3). Because macronutrients are coupled with various biochemical and cellular constituents, the ratios of these elements remained relatively constant compared to the potentially decoupled resource stoichiometry changes in the surrounding environment that were induced by warming [1], [39].

In contrast, tight and significant associations between ratios of soil C∶P and microorganism C∶P were obtained with the fitted curves of the power model (Fig. 3), which seems contradictory to the results described above. This may be explained by soil microorganisms regulating the equilibrium between the elemental composition of their biomass and the efficiencies of microbial nutrient assimilation and growth through ecoenzymatic interactions [26]. Further, simultaneous N and P enrichment (by photosynthetic biota) produces strongly positive, synergistic responses in ecosystems [16]. This synergistic effect can aid in maintaining “Redfield-like” ratios in soil microorganisms [39], and may also have served to regulate the stoichiometric balance of soil microbial C and P acquisition for the warmed wetland soils in this study. This is also in line with general ecological theory on the elemental stoichiometries of microbial biomass and environmental availability relative to microbial nutrient assimilation and growth [1], [32]. For example, increases in N concentration in biomass are usually accompanied by increases in P uptake for vascular plants [11]. This suggests that the synergistic function of C-P acquisition is an important mechanism underlying the C∶P stoichiometric balance for soil microorganisms in wetland ecosystems subjected to warming.

Field N and P fertilization in another study showed that higher values of *H_N∶P_* in grasses were significantly associated with higher species dominance and stability [2]. In this study, the homeostatic regulation coefficient for C∶P stoichiometry in the soil microorganism community vs soil HLOC∶DRP ratios (i.e., *H_C∶P_*) was lower under the warmed treatment than the ambient treatment (Fig. 3). In ecoenzymatic stoichiometry theory [26], [32], the relative availability of hydrolyzed P via ecoenzymes (such as phosphatase) in soils relative to C in the microbial community composition was higher under experimental warming than in the control treatments. Ecosystem function and stability may have been impacted in the warmed soil columns in this study, with a negative allometric relationship to soil microorganism *H_C∶P_*. Our previous research has shown that a significant increase in soil microorganism biomass measured by total phospholipid fatty acids (PLFAs) occurred under warming [21], which is line with the data in Table 1; however, bacterial abundance in the soil tended to decrease along with an increase in the ratio of fungi∶bacteria [21], [40], [41]. Furthermore, relatively high abundances of fungi in soil ecosystems increase the secretion of P-hydrolyzing enzymes in soils [21], [42], [43]. Meanwhile, functional gene array data verified that soil P utilization genes (such as polyphosphate kinase and exopolyphosphate) were also enhanced by warming [27], leading to increases in soil microbial P pools (Table 1) and speeding up nutrient cycling processes.

In a 13 d laboratory incubation test of YT wetland sediment (Text S3), lower dissolved oxygen (DO) concentrations in overlying water (Fig. S2) and higher ferrous iron (Fe^2+^) concentrations in sediment (Fig. S3) were found for sediment sampled from the warmed column compared to the control treatment. This indicates that the redox-enhanced sediment under the warmed treatment could increase dissimilatory reduction of P-bound metal oxides and the decomposition of organic matter, liberating P from sediment aggregates. This may explain the relatively high levels of P in porewater and potential P flux under warming (Table 2). In addition to stimulating net primary production, warming could significantly increase both soil respiration and the associated C-degrading microbial genes [27], [44], increasing the C flux from terrestrial ecosystems to the atmosphere in the global C cycle [8].

Results from our previous investigation [21], coupled with the data reported here, lend further support to the use of *H_C∶P_, H_C_,* and *H_P_* as ecological ‘indicators’ (Fig. 3, 1, & 2, respectively). In other words, *H_C∶P_* values in the soil microorganism community in this study were positively correlated with C-P-related ecological function and stability. Because C∶P stoichiometric homeostasis is associated with synergistic C-P acquisitions (as discussed above), a decreased *H_C∶P_* value under warming conditions (Fig. 4) means that C biodegradation and P mineralization exceeded microorganism utilization of these elements, leading to increases in both C loss and P export. This has a “double-negative” effect on climate change and water quality.

We did not find a consistent relationship between *H_C∶P_* values in warmed vs. control treatments in terms of LOC∶DRP and MLOC∶DRP ratios (Table 4). Soil C losses in this study were traced to the recalcitrant fraction, while pools of labile C were not depleted (Table 1). Carbon availability from HLOC for the soil microorganism assemblage and metabolism is generally higher than for the rest of the C sources [31]; therefore the index of HLOC is relatively sensitive to C dynamics for microorganism acquisition. Further mechanistic investigations are needed to establish direct links between the destabilization of soil organics and soil microorganism community dynamics in order to better assess the relationship between the availability of different forms of C to C∶P stoichiometric dynamics and their ultimate effects on *H_C∶P_*.

### Does homeostasis link to soil phosphorus flux?

Efforts to predict P flux or P dynamics from the soil biosphere to aquatic ecosystems are becoming increasingly important in light of concerns related to eutrophication, climate change, and other anthropogenic impacts [19], [20], [45], [46]. Statistics-based modeling integrates various parameters, such as soil physicochemical properties, regional meteorology, and anthropogenic activities [19], [20], [23], and can be used to predict P flux under IPCC climate scenarios [4]. However, these models are insufficiently related to the ecological forcing of microorganisms in the soil micro-ecosystem, even though such microorganisms essentially mediate and drive Earth's biogeochemical cycles [13], [27]. We found that potential P flux (Table 2) was significantly related to n-*H_C∶P_* according to a negative allometric model (Fig. 4), verifying the hypothesis that a n-*H_C∶P_* index may be useful as an ecological tool to predict P flux. Although C∶P stoichiometric dynamics alone can provide some insight into P dynamics in an ecosystem [5], [39], the n-*H_C∶P_* term (as outlined in equation 4) comprehensively incorporates both soil C and P availability and soil microorganism nutrient acquisition under the principle of stoichiometric homeostasis [1]. The n-*H_C∶P_* response mirrors mechanisms related to the metabolic theory of ecology [5], [6] in that the soil micro-environment of wetland ecosystems responds positively (albeit via a negative allometric relationship) to temperature increases.

According to the derived models of potential P flux to n-*H_C∶P_* (Fig. 4), lower n-*H_C∶P_* values in this study indicate higher P flux, which is fundamentally consistent with the ecological interpretation of *H_C_* (Fig. 1), *H_P_* (Fig. 2), and *H_C∶P_* (Fig. 3) between the two treatments. Although regression models differed, the ecological role of n-*H_C∶P_* in this study is essentially identical in its nature to the linear and positive relationships between community *H* and ecological production and stability in a Mongolian grassland [2]. The consistency of these results suggests that although diverse factors may affect P dynamics, n-*H_C∶P_* may be a useful ecological tool for assessing potential P flux. Notably, the mathematic formulations between n-*H_C∶P_* and potential P flux were identical between the two experimental treatments (Fig. 4), suggesting that the feasibility of n-*H_C∶P_* to quantitatively assess potential P flux might be mainly determined by the features of soil C-P stoichiometric homeostasis itself.

It should be noted that both n-*H_C∶P_* and potential P flux are calculated using one of the same parameters; namely, soil porewater DRP, and are therefore inherently interrelated to some extent. However, n-*H_C∶P_* (Eq 4) is computed using a multifactor equation linking soil C∶P ratio (in form of soil HLOC: soil porewater DRP), soil microorganism biomass C∶P ratio, and the associated homeostatic coefficient (*H_C∶P_*). Similarly, changes in potential P flux (Eq 5) were jointly determined by differences in the concentration of DRP in soil porewater vs. overlying water, by the quantity of overlying water and the area of topsoil in the wetland column, and by the overlying water replacement time interval. Moreover, values of these two indices were distinctly influenced by experimental warming (Table 2, Fig. 3). Therefore, the strong, negative allometric relationship observed between these indices (Fig. 4) is indeed an indication of the potential ecological management role of n-*H_C∶P_* in assessing potential P flux, and is not be expected outright based only on co-variation arising from their common incorporation of a soil porewater DRP parameter.

The stoichiometric parameter S_C/P_ (or S_C/N_) has been defined as a scalar for the relative availability of hydrolyzed P (or N) in relation to microbial community composition [32], [47]. Utilizing this S_C/P_ (or S_C/N_) metric, an allometric biogeochemical equilibrium model was developed to predict microbial growth efficiency (GE) from elemental C∶N and C∶P ratios in biomass (B_C/N_ and B_C/P_, respectively) and environmental substrate sources (L_C/N_, L_C/P_), integrated with ratios of ecoenzymatic activities (EEAs) that mediate C, N, and P acquisition (EEA_C/N_, EEA_C/P_) [32]. Although the intended prediction outcomes of these approaches differ, the nature of the relationship between microbial n-*H_C∶P_* and sediment potential P flux is fundamentally similar to the use of the S_C/P_ indictor, because both scalars reflect how stoichiometric balance/interaction is regulated by environmental signals. Further, they can also both be viewed in terms of an energy landscape (i.e., C) that directs the availability and flow of resources (e.g., N and P). Therefore, our experimental data and the measurement of soil n-*H_C∶P_* might be practical for comparative investigations of soil P dynamics across a variety of wetland ecosystems. This n-*H_C∶P_* method offers a new alternative for the measurement and understanding of soil P biogeochemical cycling, comparable to current methodologies that utilize biochemistry [37], [42], molecular biology [21], [27], [43], [46], isotopic tracing [48], and mechanistic models [19], [23], [45]. To the best of our knowledge, this is the first study of integrated C-P stoichiometric homeostasis to clearly demonstrate the linkage between soil n-*H_C∶P_* and potential P flux in a freshwater wetland ecosystem.

Undoubtedly, aquatic plants are another primary pool for P biogeochemical cycling, because 12%–85% of P in water may be immobilized by wetland plants [48], [49]. However, addressing the influence of aquatic plants on n-*H_C∶P_* and other parameters was beyond the purpose of this study, and thus they were temporarily excluded from the soil columns during the course of the experiment. The involvement of aquatic plants in influencing soil C∶P stoichiometric homeostasis and associated variations in n-*H_C∶P_* thus requires further investigation.

Although soil respiration at the global scale responds positively to air temperature, in the long term warming may also mobilize older stored carbon, potentially resulting in higher carbon inputs to the soil biosphere [8]. If so, labile P forms would be ‘returned’ to the soil-microorganism complex under warming according to principles of ecoenzymatic stoichiometry [26] and mechanisms of synergistic C-P acquisition. Such an outcome would be somewhat inconsistent with the near-term decrease in soil C stock (Table 1) and increase in potential P flux (Table 2) we observed in the current microcosm warming experiment. Thus, it will also be necessary to further explore the linkage between n-*H_C∶P_* and P flux under long-term warming.

In summary, microcosm warming impaired the stoichiometric homeostatic ability (*H*) of soil microorganisms to regulate P and C biogeochemical processes. This resulted in a “double negative” effect for wetland ecosystem services: increasing their potential for P export and enhancing their role as a recalcitrant organic C source. Moreover, the specialized homeostatic regulation ability (n-*H_C∶P_*, as a function of the C∶P ratio in the form of HLOC to DRP in soil porewater relative to the C∶P ratio in soil microbial biomass) of soil microorganisms was inversely linked to potential P flux at the soil-water interface. Based on our results, we advocate further use of homeostatic regulation indices (*H*) as novel ecological tools for assessing potential P flux and other biogeochemical dynamics in the face of global climate change.

## Supporting Information

Figure S1 **The design of the experimental wetland microcosm system setup by using independently monitored water bath jackets under the current climate condition (Left: Ambient temperature, Control) and the warming climate condition (Right: Ambient temperature +5°C, Warmed treatment).**
(DOC)

Figure S2 **Dynamics of dissolved oxygen (DO) concentration in overlying water during 12 d of sediment incubation.** Sediment samples were collected in July 2010 from YaTang riverine wetland (YT) under control (ambient temperature) and warmed (ambient temperature +5°C) treatments in microcosm experiment. Error bars show ± SD. The differences between control and warmed treatments were tested by Student's *t*-test for each sampling point, indicated by * *p*<0.05, ** *p*<0.01.
(DOC)

Figure S3 **Dynamics of ferric iron (Fe^3+^) and ferrous iron (Fe^2+^) concentration in sediment measured during a 13-d laboratory incubation for YaTang riverine wetland (YT) sediment samples under control (ambient temperature) and warmed (ambient temperature +5°C) treatments.** Error bars show ± SD. The differences between control and warmed treatments were tested by Student's *t*-test for each sampling point, indicated by * *p*<0.05, ** *p*<0.01.
(DOC)

Table S1 **Details of the six selected wetland sites used in the study.**
(DOC)

Table S2 **Select physico-chemical parameters of 20-cm depth soil samples collected from the JinHu wetland (JH), XiaZhuhu wetland (XZ), YaTang riverine wetland (YT), XiXi national wetland park (XX), BaoYang riverine wetland (BY), and ShiQiuyang multipond wetland (SQ) in May 2008.**
(DOC)

Text S1 **Microcosm configuration.**
(DOC)

Text S2 **Wetland column preparation and field sampling.**
(DOC)

Text S3 **Laboratory incubation for sediment oxygen demand and reducing capability.**
(DOC)
