# Supplementary material for: Linking Stoichiometric Homeostasis of Microorganisms with Soil Phosphorus Dynamics in Wetlands Subjected to Microcosm Warming
Source: PLoS One. 2014 Jan 27;9(1):e85575. doi: 10.1371/journal.pone.0085575 (PMC3903482; doi:10.1371/journal.pone.0085575)
Supplement: Figure S1 — The design of the experimental wetland microcosm system setup by using independently monitored water bath jackets under the current climate condition (Left: Ambient temperature, Control) and the warming climate condition (Right: Ambient temperature +5°C, Warmed treatment). (DOC) [file pone.0085575.s001.doc]

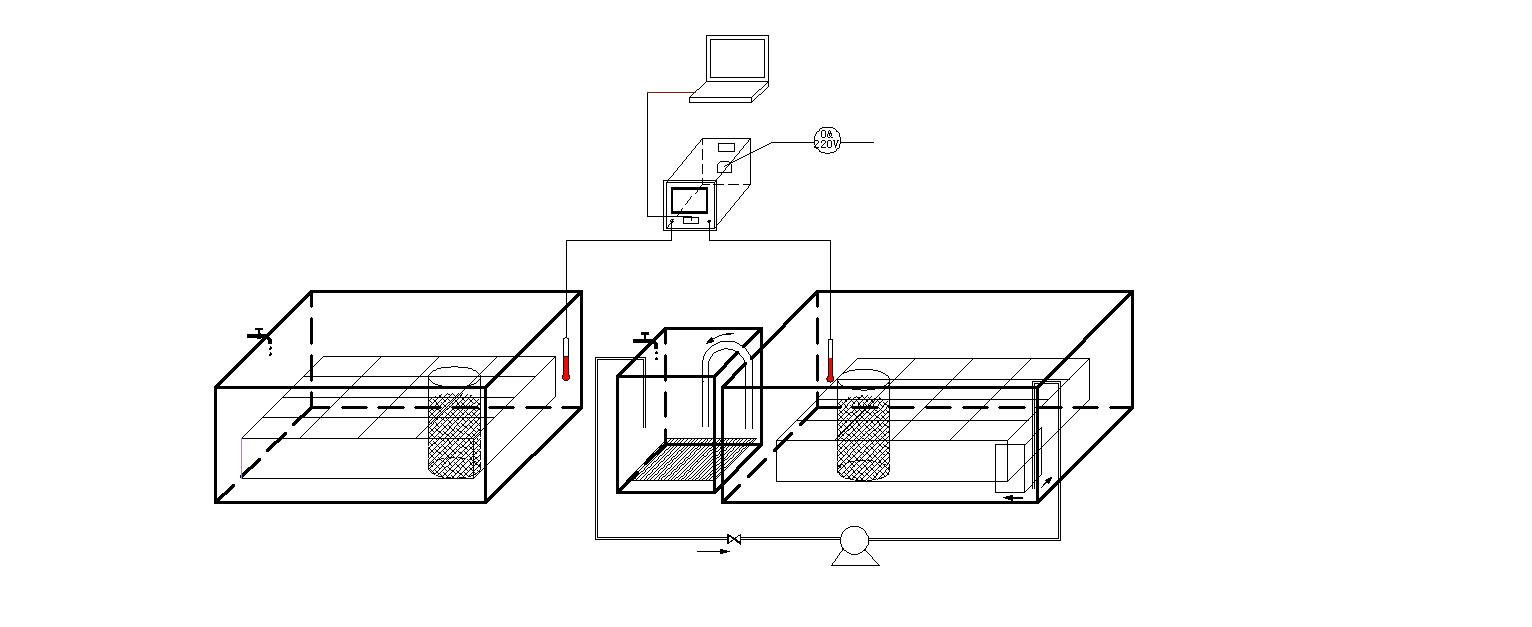


Tap water

**Ambient temperature (Control)**

**Ambient temperature + 5^o^C (Warmed)**

Heater

Temperature probes

Power

Computer

Stand

Wetland columns

Pump

Wire

Stainless steel box

Pipe

U-tube

Accessorial

tank

Controller

100 cm

100 cm

40 cm

Sun

**Water**

**Figure S1.** The design of the experimental wetland microcosm system setup by using independently monitored water bath jackets under the current climate condition (Left: Ambient temperature, Control) and the warming climate condition (Right: Ambient temperature +5^o^C, Warmed treatment).
