# Supplementary material for: Linking Stoichiometric Homeostasis of Microorganisms with Soil Phosphorus Dynamics in Wetlands Subjected to Microcosm Warming
Source: PLoS One. 2014 Jan 27;9(1):e85575. doi: 10.1371/journal.pone.0085575 (PMC3903482; doi:10.1371/journal.pone.0085575)
Supplement: Figure S3 — Dynamics of ferric iron (Fe3+) and ferrous iron (Fe2+) concentration in sediment measured during a 13-d laboratory incubation for YaTang riverine wetland (YT) sediment samples under control (ambient temperature) and warmed (ambient temperature +5°C) treatments. Error bars show ± SD. The differences between control and warmed treatments were tested by Student's t-test for each sampling point, indicated by * p<0.05, ** p<0.01. (DOC) [file pone.0085575.s003.doc]

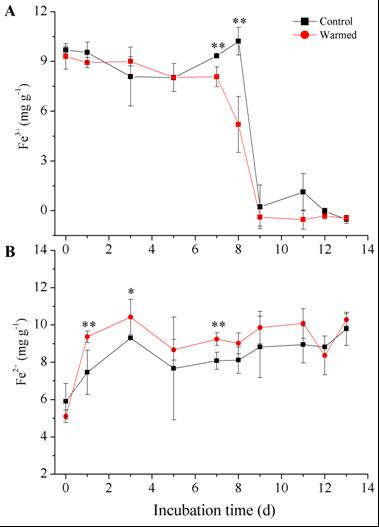


**Figure S3** Dynamics of ferric iron (Fe^3+^) and ferrous iron (Fe^2+^) concentration in sediment measured during a 13-d laboratory incubation for YaTang riverine wetland (YT) sediment samples under control (ambient temperature) and warmed (ambient temperature + 5^o^C) treatments. Error bars show ± SD. The differences between control and warmed treatments were tested by Student’s *t*-test for each sampling point, indicated by * *p* < 0.05, ** *p* < 0.01.
