# Supplementary material for: Linking Stoichiometric Homeostasis of Microorganisms with Soil Phosphorus Dynamics in Wetlands Subjected to Microcosm Warming
Source: PLoS One. 2014 Jan 27;9(1):e85575. doi: 10.1371/journal.pone.0085575 (PMC3903482; doi:10.1371/journal.pone.0085575)
Supplement: Table S1 — Details of the six selected wetland sites used in the study. (DOC) [file pone.0085575.s004.doc]

**Table S1. Details of the six selected wetland sites used in the study**

| Name | County | Latitude and longitude | Main wetland use | Annual mean water depth, m | Flow rate,  m min^-1^ | Dominant macrophytes |
| --- | --- | --- | --- | --- | --- | --- |
| JinHui (JH) | ShaoXing | 120°33'32"E, 30°01'58"N | Water reservoir | 2.50 | 0.0-0.05 | *Trapa bispinosa, Alternanthera philoxeroides, Trapa* spp, *Arundo dona*, *Arundo donax* |
| XiaZhuhu (XZ) | DeQing | 120°02'54"E, 30°31'28"N | Tourism and aquaculture | 1.50 | 0.0-0.12 | *Trapa bispinosa, Alternanthera philoxeroides, Trapa* spp, *Arundo dona*, *Arundo donax* |
| YaTang riverine wetland (YT) | TongXiang | 120°29'13"E, 30°43'15"N | Mixed use | 0.80 | 0.0-1.02 | *Trapa bispinosa, Alternanthera philoxeroides, Trapa* spp, *Arundo dona*, *Arundo donax* |
| XiXi National Wetland Park (XX) | HangZhou City | 120°03'59"E, 30°16'23"N | Tourism | 0.85 | 0.0-0.10 | *Phragmites communis, Trapa* spp, *Acorus calamus, Sagittaria sagittifolia, Phragmites communis,*  *Miscanthus floridulus* |
| BaoYang riverine wetland (BY) | ChangXing | 119°54'24"E, 31°04'31"N | Water reservoir | 0.68 | 0.0-1.32 | *Trapa bispinosa, Alternanthera philoxeroides, Trapa* spp, *Arundo dona*, *Arundo donax* |
| ShiQiu multipond wetland (SQ) | JiaXing City | 120°41'31"E, 30°53'55"N | Water reservoir | 1.20 | 0.0-1.68 | *Phragmites communis, Trapa* spp, *Acorus calamus, Sagittaria sagittifolia, Phragmites communis,*  *Miscanthus floridulus* |
