# Supplementary material for: Linking Stoichiometric Homeostasis of Microorganisms with Soil Phosphorus Dynamics in Wetlands Subjected to Microcosm Warming
Source: PLoS One. 2014 Jan 27;9(1):e85575. doi: 10.1371/journal.pone.0085575 (PMC3903482; doi:10.1371/journal.pone.0085575)
Supplement: Table S2 — Select physico-chemical parameters of 20-cm depth soil samples collected from the JinHu wetland (JH), XiaZhuhu wetland (XZ), YaTang riverine wetland (YT), XiXi national wetland park (XX), BaoYang riverine wetland (BY), and ShiQiuyang multipond wetland (SQ) in May 2008. (DOC) [file pone.0085575.s005.doc]

**Table S2.** Select physico-chemical parameters of 20-cm depth soil samples collected from the JinHu wetland (JH), XiaZhuhu wetland (XZ), YaTang riverine wetland (YT), XiXi national wetland park (XX), BaoYang riverine wetland (BY), and ShiQiuyang multipond wetland (SQ) in May 2008.

| Sampling site | pH | Total organic C  (g kg^-1^) | Total N  (g kg^-1^) | Total P  (mg kg^-1^) |
| --- | --- | --- | --- | --- |
| JH | 7.2 | 25.5 | 1.89 | 579.2 |
| XZ | 7.3 | 64.7 | 4.32 | 906.2 |
| YT | 7.4 | 114.3 | 6.81 | 2530.2 |
| XX | 7.4 | 32.6 | 3.87 | 520.6 |
| BY | 7.1 | 39.3 | 2.40 | 833.4 |
| SQ | 7.3 | 14.6 | 1.45 | 345.7 |
