# Supplementary material for: Linking Stoichiometric Homeostasis of Microorganisms with Soil Phosphorus Dynamics in Wetlands Subjected to Microcosm Warming
Source: PLoS One. 2014 Jan 27;9(1):e85575. doi: 10.1371/journal.pone.0085575 (PMC3903482; doi:10.1371/journal.pone.0085575)
Supplement: Text S1 — Microcosm configuration. (DOC) [file pone.0085575.s006.doc]

**Text-S1: Microcosm conﬁguration**

A microcosm (Fig. S1) simulating climate warming at a minute scale for both daily and seasonal scenarios was developed by using independently monitored water bath jackets for this study. The microcosms consisted of four major components, a storage section, a heating section, a water circulation section, and a real-time controlling section. The storage section was composed of two stainless steel incubation boxes: one was for the current ambient temperature treatment (Control), and the other was for the +5^o^C increased temperature treatment (Warmed). The real-time controlling section was composed of a computer (HP a6315cn), a custom-built controller (TZ2008, Jiaxing China), digital temperature probes (NB 407-25a, China), and lab-designed software (C^++^ language). The temperature probes, heater, and pump were programmed through the custom-built controller by the computer. With the help of the software, the temperatures in both incubation boxes were continuously recorded and the differences in the box temperatures were compared by digital probes at two-minute intervals. The temperature difference between the two incubation boxes was set at 5^o^C±1^o^C. Thereafter, the custom-built controller simultaneously turned the heater and the pump on or off when the instantaneous temperature difference was lower than 4^o^C or over 6^o^C, respectively. Except for the computer and the controller, the rest of the microcosm components were set up outdoors in May 2008. This novel microcosm offers a higher resolution temperature difference (based on minute scale), simulating a more realistic warming condition, compared to previously reported temperature manipulation experiments.
