# Supplementary material for: Linking Stoichiometric Homeostasis of Microorganisms with Soil Phosphorus Dynamics in Wetlands Subjected to Microcosm Warming
Source: PLoS One. 2014 Jan 27;9(1):e85575. doi: 10.1371/journal.pone.0085575 (PMC3903482; doi:10.1371/journal.pone.0085575)
Supplement: Text S2 — Wetland column preparation and field sampling. (DOC) [file pone.0085575.s007.doc]

**Text-S2: Wetland column preparation and field sampling**

Transparent PVC wetland columns (45.0 cm in height and 10.0 cm in internal diameter) were prefabricated before sampling. Each wetland column was designed to be filled with 20 cm of fresh soil and 20 cm of the corresponding overlying water. The field sampling was conducted in May 2008. Individual samples of soil cores from 0-15 cm from the surface were collected with a stainless steel column sampler. After most of the larger visible roots, stones, and macrobenthos were removed from the surface, the soil (mixed with its zoobenthos community, mainly including Olisochaeta, Crustacea and Mollusca) sample was divided into a 0-5 cm topsoil and 5-15 cm sub-soil manually. Then the 5-15 cm sub-soil was first transferred into the 5-20 cm wetland column, after which the 0-5 cm top-soil was refilled carefully to the remaining 0-5 cm column space of soil layer. After 20-cm-depth soil refilling, each column was filled with 20 cm of the ambient overlying water. Although this soil refilling in layers is not rigorously classified as intact cores, these experimental wetland columns basically exhibit the biogeographic features of the topsoil micro-environment for these sampling sites. All of the wetland columns (with six replicates for each wetland site) were shipped back to the laboratory within three hours where three replicates of each wetland sample were placed inside each of the two incubation boxes. For porewater sampling, an orbicular Teflon tube (TCN-350 Nanjing: 1.0 mm in diameter, 1.0 µm in aperture) was horizontally embedded into the soil in each column at a depth of 5 cm. Some of floating-leaved (e.g., *Lemna minor L., Trapa spp.*) and submerged (e.g., *Ceratphyllum demersum L.*) aquatic vegetation was found growing after two months of incubation.

The tested wetland ecosystems are located in floodplain areas within the Yangtze River delta, and the average hydraulic retention time generally varies from a few weeks to months due to changes in seasonal rainfall and frequency of water utilization. To standardize the experimental operation for simulation incubations, we manually changed overlying water once every two months after the first six months of incubation. Therefore, water samples were taken from the microcosm columns once every two months. One hundred mL of overlying water sample was sampled from each column 5 cm below the water surface with a syringe and was acidified to pH 2 by adding diluted HCl. Porewater for each wetland column was collected in the orbicular Teflon tube by a standard 50-mL syringe (-0.05 MPa vacuum pressure) twice on each sampling day, then combined for storage and analysis. Before sampling, 0.10 mL of 4.0 mol L^-1^ HCl was injected into each syringe to reduce the pH of the expected 50 mL water sample to 2 in order to preserve the sample. After water samples were collected, the overlying water remaining inside the column was carefully emptied into a plastic graduated flask by hand and then refilled with spring water (Nongfu Food Company, China) up to 20 cm in depth so that a 60-day period would mimic the general hydraulic activity for these field wetlands. Total concentrations of phosphorus, organic carbon, calcium, magnesium, potassium, sodium, and pH for this commercial spring water are recorded as 0.02 mg L^-1^, 8.5 mg L^-1^, 5.4 mg L^-1^, 0.63 mg L^-1^, 0.45 mg L^-1^, 0.97 mg L^-1^, and 7.3 on average, respectively. Although replacing overlying water uniformly by using the commercial spring water could diminish the effect of original nutrient loading on water column productivity at each of sampling sites, such homogeneous operation allows evaluation of the relative intensity of soil-water P and C turnover in wetland columns subjected to experimental warming in comparison to ambient temperature treatments. In addition, approximately 100 g of fresh soil in a 0-5 cm layer was manually collected for each column using a lab spoon in July and November 2010, and in March 2011 during incubation, before refilling the column with water. All water and soil samples were frozen at -15^o^C prior to further analysis.
