# Supplementary material for: Linking Stoichiometric Homeostasis of Microorganisms with Soil Phosphorus Dynamics in Wetlands Subjected to Microcosm Warming
Source: PLoS One. 2014 Jan 27;9(1):e85575. doi: 10.1371/journal.pone.0085575 (PMC3903482; doi:10.1371/journal.pone.0085575)
Supplement: Text S3 — Laboratory incubation for sediment oxygen demand and reducing capability. (DOC) [file pone.0085575.s008.doc]

**Text S3: Laboratory incubation for sediment oxygen demand and reducing capability**

Sediments collected from YT wetland columns in the summer (Jul 2010) were chosen for laboratory assays of sediment reducing capability. This site was chosen because the impact of experimental warming on YT wetland was the most significant among three tested wetlands in terms of sediment redox potential shifts. In addition, the summer samples from this site had relatively high microbial activities, where the effects of elevated temperature and associated low redox potential were more prominent than in other sampling seasons. Two parameters, i.e., dynamics of dissolved oxygen (DO) concentration in overlying water, and dynamics of ferric and ferrous iron (Fe^3+^ and Fe^2+^, respectively) concentration in sediment, were evaluated for sediment reducing capacity in this study.

The 0-5 cm top sediment cores were collected using thin-walled plastic core tubes. For measurement of the dynamics of dissolved oxygen (DO) concentration in overlying water, each core was transferred to a 5-cm (inner diameter), 20 cm-long glass container and filled with a 10-cm oxygen-saturated water column above the core. After flushing with N_2_, the glass container was closed tightly using stoppers, and then incubated at a constant temperature of 25 ^o^C in the dark. The dissolved oxygen (DO) concentration in overlying water was measured using a DO meter (HQ30d, HACH Corporation, American) at each sampling time point. A lower level of DO in overlying water indicates higher levels of sediment reducing capability.

For measurement of dynamics of ferric iron (Fe^3+^) and ferrous iron (Fe^2+^) concentration in sediment, a sediment suspension, prepared by re-suspending 10 g of wet sediment from a homogenized 0-5 cm top sediment core in 50 mL distilled water, was incubated at a constant temperature of 25^o^C in the dark. Acetate substrate (20 mmol L^-1^) was first added three days before the beginning of the assay as a carbon source to stimulate the activity of microorganisms. After that, the electron acceptor (FeC_6_H_5_O_7_·H_2_O, 200 mg Fe L^-1^) and acetate substrate (20 mmol L^-1^) were added in each core in order to measure ferric iron reduction rates. For each sampling time point, 2 mL of homogeneous sample were taken, centrifuged at 500 x g for 10 min to remove sediment particles and then diluted before analysis. According to standard methods (Wei 2002), the phenanthroline spectrophotometric method was conducted for measurements of Fe(II) and Fe(III) using a continuous flow analyzer (Autoanalyzer III, BRAN+LUEBBE, Germany). The higher level of Fe(II) in sediment indicates the higher levels of sediment reducing capability.

References:

Wei F (2002). *Water and wastewater monitoring and analysis*. Environmental Science Press: Beijing, China.
